# Low-Energy Collisions of Zeeman-Decelerated NH Radicals with He Atoms

**DOI:** 10.1021/acs.jpca.2c08712

**Published:** 2023-03-08

**Authors:** Vikram Plomp, Jolijn Onvlee, François Lique, Sebastiaan Y. T. van de Meerakker

**Affiliations:** † Institute for Molecules and Materials, Radboud University, Heyendaalseweg 135, 6525 AJ Nijmegen, The Netherlands; ‡ Institut de Physique de Rennes, Université de Rennes 1, 263 avenue du Général Leclerc, 35042 Rennes CEDEX, France

## Abstract

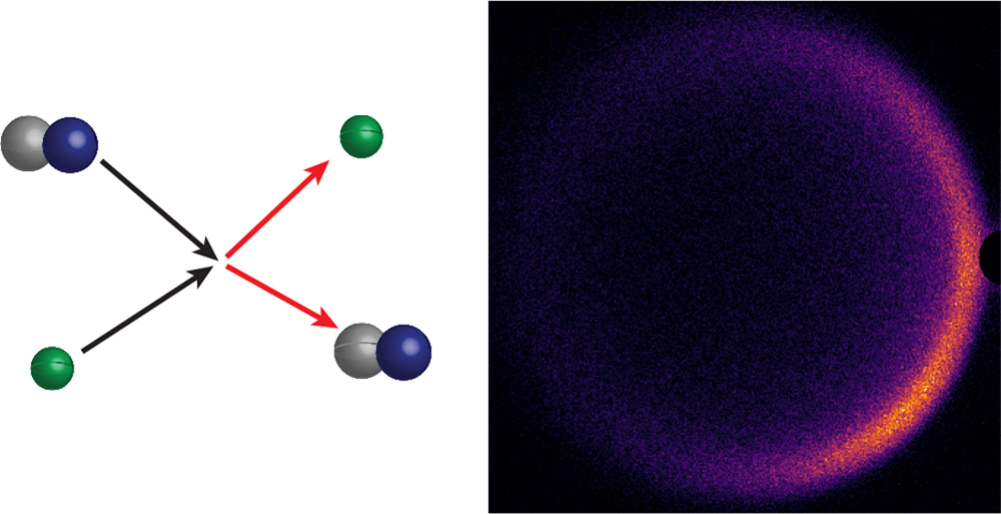

We report an experimental study of state-to-state inelastic scattering of NH (X ^3^Σ^–^, *N* = 0, *j* = 1) radicals with He atoms. Using a crossed molecular beam apparatus that combines a Zeeman decelerator and velocity map imaging, we study both integral and differential cross sections in the *N* = 0, *j* = 1 → *N* = 2, *j* = 3 inelastic channel. We developed various new REMPI schemes to state-selectively detect NH radicals, and tested their performance in terms of sensitivity and ion recoil velocity. We found a 1 + 2′ + 1′ REMPI scheme using the A ^3^Π ← X ^3^Σ^–^ resonant transition, which yields acceptable recoil velocities and is more than an order of magnitude more sensitive than conventional one-color REMPI schemes to detect NH. We used this REMPI scheme to probe state-to-state integral and differential cross sections around the channel opening at 97.7 cm^–1^, as well as at higher energies where structure in the scattering images could be resolved. The experimental results are in excellent agreement with the predictions from quantum scattering calculations which are based on an *ab initio* NH-He potential energy surface.

## I Introduction

In recent years, Stark deceleration of neutral polar molecules has emerged as a powerful method to precisely study molecular collision processes in crossed beam experiments, and at low collision energies in particular. The molecular packets that emerge from the decelerator have a tunable velocity, a high state purity, narrow transverse and longitudinal velocity spreads, and a narrow spatial spread.^1^ They serve as excellent starting points in scattering experiments, and in combination with velocity map imaging (VMI) detection techniques, they have been essential to probe scattering processes in unprecedented detail.^2^ Examples include the observation of diffraction oscillations,^3,4^ partial wave scattering resonances at low collision energies,^5−7^ and rotational product pair correlations in bimolecular inelastic scattering processes.^8,9^

By its nature, the Stark deceleration technique is only applicable to molecules that have an electric dipole moment, excluding an important class of species that are inert to electric fields. Yet, these species often have a magnetic dipole moment and can be manipulated by magnetic fields instead. Zeeman decelerators, the magnetic analogue of Stark decelerators, have been developed as well and have successfully been used to slow a variety of species and to load them into traps; see reviews in refs (10−13) and references contained within. Recently, the first successful crossed beam scattering experiments using a Zeeman decelerator have been performed, focusing on inelastic processes involving NO radicals or C atoms.^14,15^ Despite these important proof-of-principle advances, crossed beam scattering using Zeeman decelerators is still in its infancy, and the full potential of the technique to probe scattering processes has yet to be unlocked. In particular, it is important to investigate whether the technique can be used for sensitive measurements on a large variety of species and whether low collision energies are achievable.

Recently, we reported the direct Zeeman deceleration of NH (X ^3^Σ^–^)^16^ using a 2.2 m long Zeeman decelerator that was specifically designed for applications in crossed beam experiments.^17^ Here, we describe the first measurements of state-to-state inelastic integral and differential cross sections for NH radicals and He atoms using a crossed molecular beam apparatus that uses a Zeeman decelerator to prepare the initial packet of NH radicals and velocity map imaging to detect the products, extending the chemical diversity of this type of experiment to the realm of molecular radicals. Furthermore, we tested various resonance-enhanced multiphoton ionization (REMPI) schemes to (i) state-selectively detect NH radicals with high sensitivity and (ii) limit the recoil velocity imparted to the ion upon ionization to minimize blurring of scattering images measured using VMI. We identified a new two-color 1 + 2′ + 1′ REMPI scheme employing the A ^3^Π ← X ^3^Σ^–^ resonant transition and subsequent ionization through a 2-photon resonant transition to a yet unidentified electronic state. This REMPI scheme was found to offer reduced ion recoil velocities and to be more than an order of magnitude more sensitive than the more commonly used 2 + 1 REMPI scheme via the D ^3^Π state. Using this detection scheme, we studied the *N* = 0, *j* = 1 → *N* = 2, *j* = 3 inelastic scattering channel for NH + He collisions at energies between 90 and 427 cm^–1^ and probed the threshold behavior of the scattering cross sections around the energetic opening at 97.7 cm^–1^. Our experimental findings are in excellent agreement with the predictions from quantum scattering calculations which are based on the recent *ab initio* NH-He potential energy surface by Ramachandran et al.^18^

The NH radical has been identified as a species of primary interest in the physical chemistry and cold molecules research communities.^19−26^ At high energies, a large body of state-to-state scattering experiments have been conducted involving NH and a variety of scattering partners.^27−29^ Its 2 μ_*B*_ magnetic moment in the X ^3^Σ^–^ electronic ground state offers a prominent Zeeman effect suitable for magnetic deceleration and trapping experiments. Trapping in the X ^3^Σ^–^ state has already been achieved via the buffer gas cooling technique^30−32^ and after Stark deceleration in the metastable a ^1^Δ state prior to optical transfer into the X ^3^Σ^–^ electronic ground state.^33^ Furthermore, NH is an important species in astrochemistry, and pronounced scattering resonances at temperatures below 150 K are theoretically predicted for collisions between NH and He atoms or H_2_ molecules.^18,34−36^ Finally, the NH molecule provides interesting prospects to control collisions or chemical reactions using externally applied electric or magnetic fields.^25,37^

## II Methods

### II.A Experimental Setup

**Figure 1:**
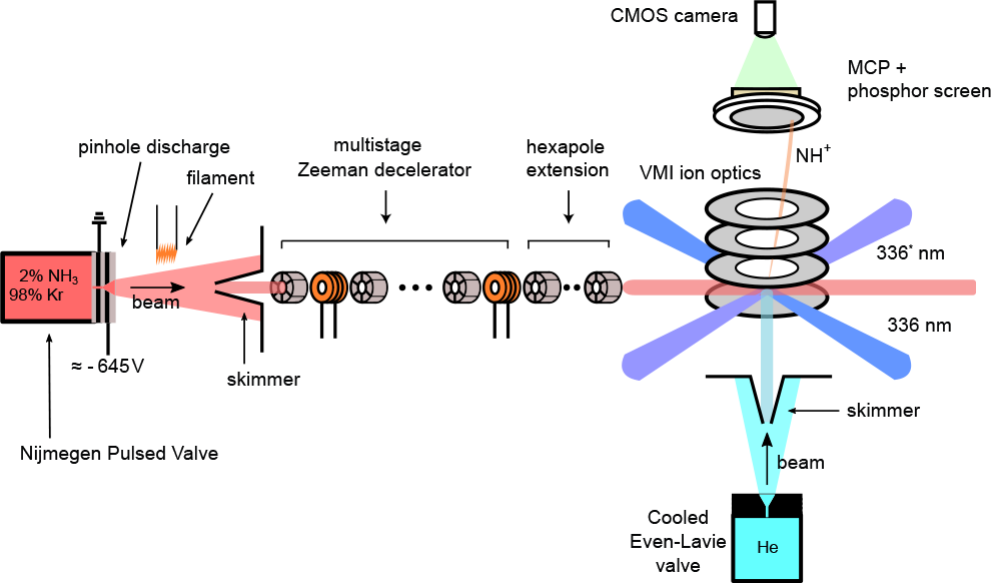
Schematic depiction of the experimental setup. NH molecules in the X ^3^Σ^–^ electronic ground state are created by an electric discharge of NH_3_ seeded in Kr. The beam is collimated by a skimmer and then passed through a Zeeman decelerator consisting of an alternating array of 100 pulsed solenoids and 101 permanent magnetic hexapoles. The NH radicals exiting the Zeeman decelerator are guided into the interaction region by a number of additional hexapoles and intercepted by a beam of helium atoms under 90 deg angle of incidence. The scattered NH radicals are detected using velocity map imaging and a variety of different REMPI schemes.

**Figure 2:**
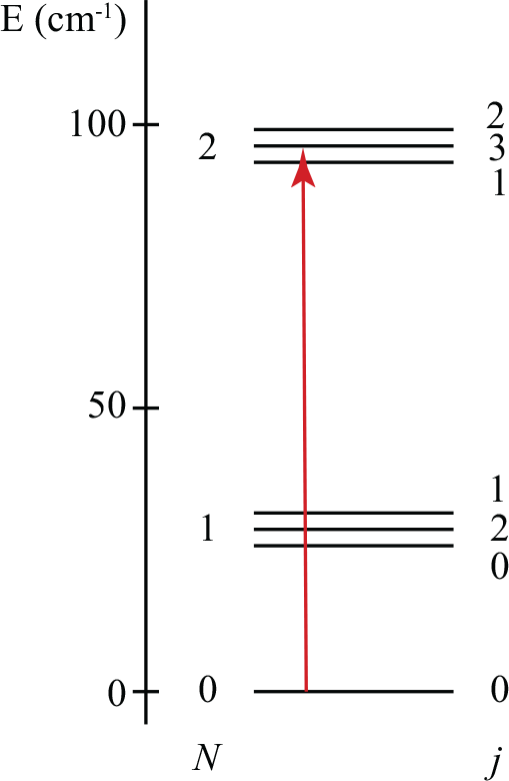
Rotational energy level diagram of NH radicals in the X ^3^Σ^–^ electronic ground state. Each rotational level is labeled by the quantum numbers *N*, *j*. The collision induced excitation channel studied in this paper is indicated by the red arrow.

The experiments are performed in the molecular beam apparatus schematically depicted in Figure 1, which was recently used to decelerate beams of O atoms, O_2_ molecules,^38^ NO (X ^2^Π_3/2_) radicals,^14^ NH radicals,^16^ and C atoms.^15^ The experiment was operated at a repetition rate of 20 Hz. A detailed description of the mechanical and electronic implementation of the decelerator, as well as details on the production and deceleration of NH radicals, are given elsewhere.^16,17^ Briefly, a molecular beam of NH (X ^3^Σ^–^) molecules with a forward velocity centered around 550 m/s is formed by an electric discharge of 2% NH_3_ seeded in krypton, using a Nijmegen pulsed valve with a discharge assembly.^39^ After the supersonic expansion, most NH radicals in the X ^3^Σ^–^ electronic ground state reside in the *v* = 0, *N* = 0, *j* = 1 rovibrational ground state (see Figure 2 for a rotational energy level scheme of the NH radical together with the Hund case (b) quantum numbers used to label the individual states). This state has a magnetic moment of 2 μ_*B*_ and splits into *m*_*j*_ = 1, *m*_*j*_ = 0, and *m*_*j*_ = −1 components in the presence of a magnetic field. Only NH radicals in the low field seeking the *m*_*j*_ = 1 component are selected by the Zeeman decelerator in the experiments. Approximately 90 mm downstream from the nozzle orifice, the molecular beam passes a 3 mm diameter skimmer and enters the Zeeman decelerator, which consists of an alternating array of 100 solenoids and 101 hexapoles.^17^ The decelerator consists of 5 modules of 20 solenoids and 19 hexapoles connected to each other using a hexapole positioned at the interface between the modules. The solenoids are made of a copper capillary through which currents up to 5 kA are pulsed. Cooling liquid is passed through the solenoids. Each solenoid is connected to an individual printed circuit board to provide the current pulses using field effect transistor (FET)-based electronics. The hexapoles consist of six commercially available arc-shaped permanent magnets. The velocity and spatial spreads of the NH packet exiting the decelerator strongly depend on the settings of the decelerator, as discussed by Plomp et al.^16^

The NH (X ^3^Σ^–^) radicals that exit the Zeeman decelerator are intersected by a secondary beam of He atoms at an angle of 90°. The He beam was produced by a cryogenically cooled Even-Lavie valve (ELV) that was temperature controlled to tune the mean velocity of the He beam. In all cases, the temporal duration of the He beam largely exceeded the temporal duration of the NH packet in the collision region. The flight from the exit of the decelerator to the interaction region amounted to about 364 mm. To reduce the loss of density during this flight, the Zeeman decelerator was extended with a hexapole array that transversally guides the molecules toward the interaction region.

The scattered NH radicals are state-selectively detected using REMPI. Depending on the experiment, different single and multicolor REMPI schemes are used (see section III.A). After the REMPI process, the NH ions are detected with a high-resolution VMI detector using a design as described by Plomp et al., to allow for accurate mapping of large ionization volumes.^40^ A repeller voltage of 2000 V is used to accelerate the ions toward a mass-gated microchannel plate (MCP) detector. Impact positions of impinging ions are recorded by a phosphor screen in combination with a CMOS camera and home-written acquisition and analysis software. For the measurement of spectra, the VMI detector is operated out of velocity focus, and the integral signal recorded by the camera is used. For the measurements of scattering images, the VMI detector is operated in velocity focus, such that 2D images that reflect the differential cross sections (DCSs) of the scattering processes are generated. For the measurement of integral cross sections (ICSs), the VMI detector is also operated in velocity focus to be able to better subtract the contribution of the initial beam (shot to shot) as well as other background signals. The measure of the ICS is then obtained by integrating the scattering signal within the relevant area of the detector. Saturation of the detector does not occur as the scattering signal is quite weak.

## III Results and Discussion

### III.A Different REMPI Schemes

To detect the scattered NH radicals using velocity map imaging, a REMPI scheme is desired that (i) allows for the state-selective detection of NH with (*N*, *j*) resolution, (ii) is sufficiently sensitive to be used in experiments employing a Zeeman decelerator, especially when considering the predicted low integral cross sections (≲1 Å^2^) of, for example, inelastic NH + He collisions,^18^ and (iii) imparts near-zero recoil velocity to the ions to optimally exploit the high inherent velocity resolution when using a Zeeman decelerator.

**Figure 3:**
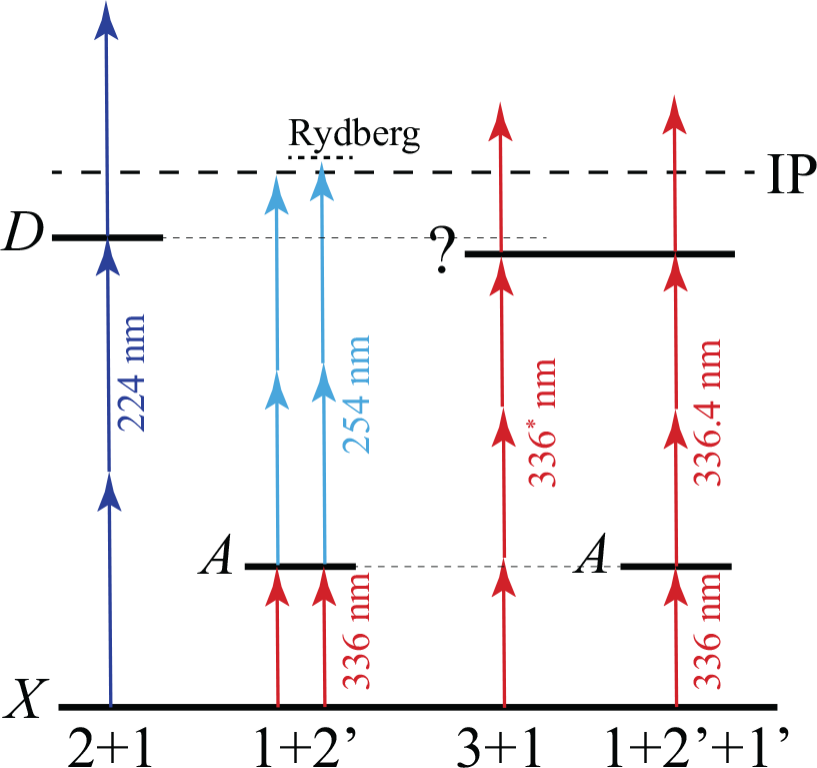
Schematic illustration of the various REMPI schemes investigated in this work to state-selectively detect NH radicals. The X ^3^Σ^–^, A ^3^Π, and D ^3^Π electronic states are indicated. The 3 + 1 and 1 + 2′ + 1′ schemes make use of a yet unidentified intermediate state, just below the energy of the D ^3^Π state. The 3 + 1 and 1 + 2′ + 1′ REMPI schemes involve a first color wavelength around 336 nm that differs slightly for the two schemes, as indicated with an asterisk. Energies of the electronic states are not drawn to scale.

A 2 + 1 REMPI scheme via the D state using photons at a wavelength around 224 nm is an established and often used REMPI scheme to state-selectively detect NH radicals.^41^ Although rather efficient, this scheme imparts a recoil velocity of about 38 m/s to the ions, significantly blurring the recorded images when VMI is used. This is inconsequential for the measurement of ICS curves in a crossed-beam experiment, but it is detrimental in measurements of scattering images. We therefore developed various alternative REMPI schemes, which we will describe in detail below, and evaluated their performance in view of the desiderata listed above. Figure 3 illustrates all schemes, whereas Table 1 summarizes the most important findings in terms of sensitivity and ion recoil velocity. The relative sensitivities of the different schemes are derived from the number of ions detected when probing the packet of NH radicals exiting the decelerator, which we list as a figure of merit in Table 1. In the following, we label spectral lines with *ΔNΔj* using the standard spectroscopic nomenclature P, Q, R, etc. to indicate the value for *ΔN* and *Δj*, where we use small and capitalized letters to label *ΔN* and *Δj*, respectively.

**Table 1:** Summary of Relevant Parameters of the Various REMPI Schemes That Were Investigated to State-Selectively Probe NH (X ^3^Σ^–^) Radicals^a^

scheme	resonant step	λ (nm)	ions/shot	recoil (m/s)
2 + 1	D ← X	224	∼100	38
1 + 2′	A ← X	336 + 254	∼5	0–7.5
3 + 1	? ← X	336*	∼2000	25
1 + 2′ + 1′	A ← X	336 + 336.4	∼2000	25

a The 3 + 1 REMPI scheme involves a first color wavelength around 336 nm that differs slightly from the wavelength used to drive the A ← X transition in the 1 + 2′ and 1 + 2′ + 1′ schemes, as indicated with an asterisk. The number of ions detected when probing the packet of NH radicals exiting the decelerator, using the experimental arrangements as specified in the text, is listed as a figure of merit to appreciate the experimental feasibility of the schemes.

**Figure 4:**
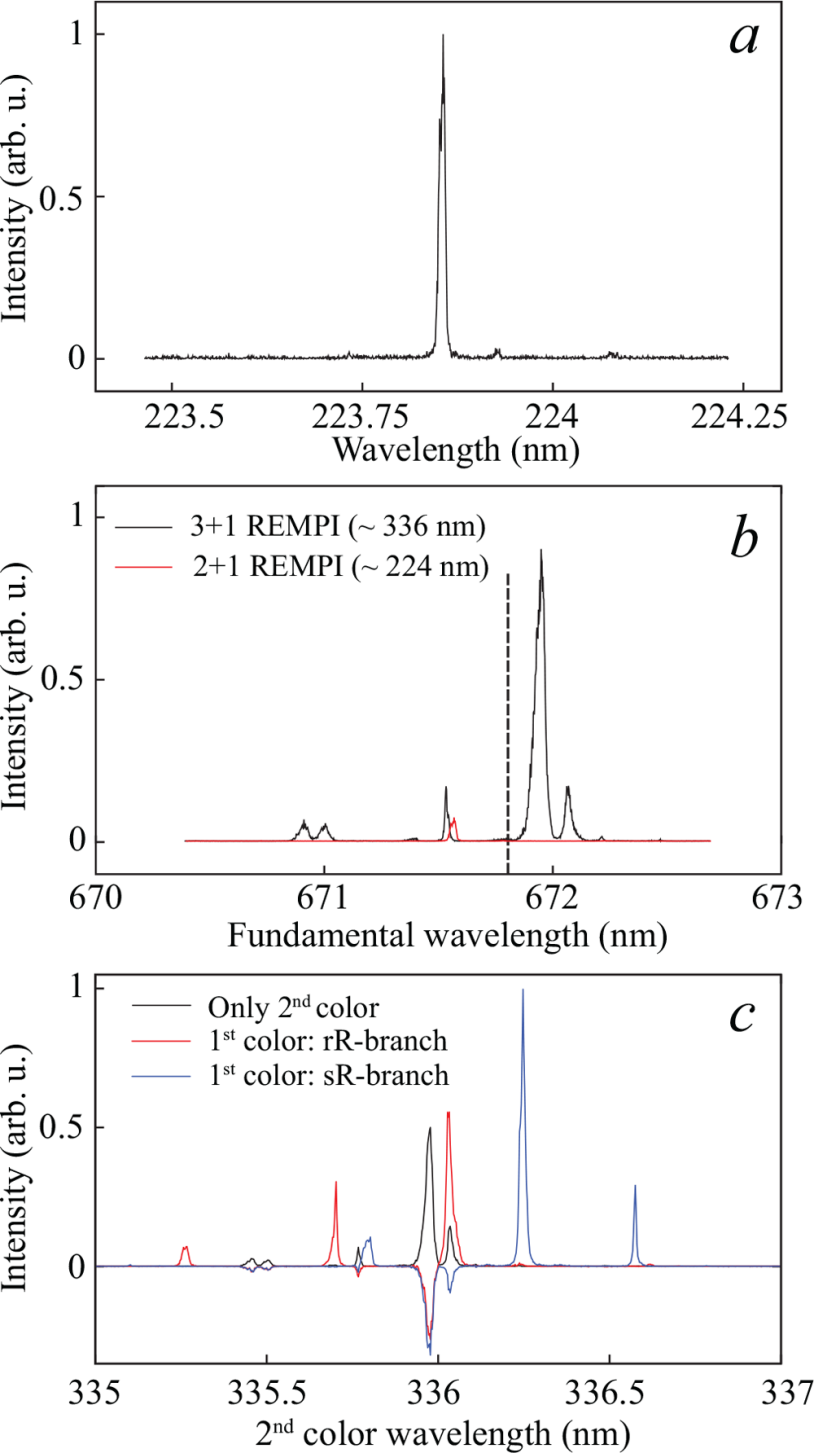
REMPI spectra probing NH (X ^3^Σ^–^) radicals exiting the Zeeman decelerator. (a) 2 + 1 REMPI spectrum using the D ← X transition. (b) 3 + 1 REMPI spectrum using an unidentified electronic transition (black), together with the 2 + 1 REMPI spectrum from panel (a). On the horizontal axis the fundamental dye laser wavelength is given before either frequency doubling or tripling. The resonance position of the A ^3^Π, *N*′ = 1, *j*′ = 2 ← X ^3^Σ^–^, *N*″ = 0, *j*″ = 1 rR transition before frequency doubling is given by the vertical dashed line. (c) 1 + 2′ + 1′ REMPI difference spectra using the rR (red) or sR (blue) branches of the A ← X transition as first color. The difference spectra are obtained by recording the two-color spectra and subsequently subtracting the spectrum recorded with the second color only (black).

To probe the performance of the well-known 2 + 1 REMPI scheme via the D state, we measured a REMPI spectrum of the packet of NH radicals that exit the decelerator with the He beam switched off. The 224 nm light is generated by frequency tripling the output of a dye laser (LiopTec), pumped using the second harmonic of a Nd:YAG laser (Innolas SL 1500). Typically, a 7 mm diameter laser beam with a pulse energy of 2.3 mJ in a 5 ns pulse is used that is focused in the interaction region using a spherical lens with 450 mm focal length. The resulting spectrum is shown in Figure 4(a). Clearly, the vast majority of the NH radicals resides in the *N* = 0, *j* = 1 rotational state, and we estimate the population in higher rotational states with a typical ratio (*N* = 0):(*N* = 1):(N = 2) ∼ 1:0.03:0.002. When the laser is tuned to the strongest peak, typically 100 ions/shot are detected.

Recently, we reported an alternative 1 + 2′ REMPI scheme.^16^ In this scheme, we first resonantly excite NH to the A ^3^Π state via the strong A ← X transition around 336 nm. The NH radicals are then ionized through the absorption of 2 photons at a wavelength around 254 nm that are provided by a second tunable dye laser. The wavelength of this ionization laser can be tuned to the ionization threshold, offering a direct route to recoil-free detection of NH. The light of the first color is generated by frequency doubling the output of a dye laser (LiopTec), pumped using the second harmonic of a Nd:YAG laser (Innolas SL 600), yielding a 5 mm diameter laser beam with 5 ns pulse width. About 10 mJ pulse energy (unfocused) was used to ensure saturation of the A ← X transition. The second color is produced by pumping a LiopTec dye laser by the second harmonic of an Innolas SL 1500 Nd:YAG laser. The output of this laser is frequency tripled to generate laser radiation at wavelengths around 254 nm. Typically, a 7 mm diameter laser beam with a pulse energy of 6.5 mJ in a 5 ns pulse is used that is focused in the interaction region using a spherical lens with 470 mm focal length. The delay between the two pump lasers was adjusted to make sure that both lasers intercept the interaction region within the 449 ns lifetime of the A ^3^Π state.^42^ In agreement with our earlier findings,^16^ although recoil-free detection of NH is possible, the ionization step is found to be rather inefficient. The ionization efficiency can be enhanced by more than an order of magnitude if the frequency of the ionization laser is blue-detuned by about 600 cm^–1^ with respect to the ionization threshold, which we speculatively attribute to autoionization of a Rydberg state.^16^ This more efficient ionization pathway is accompanied by a small ion recoil velocity of a few m/s, such that this 1 + 2′ REMPI scheme offers a trade-off between ionization efficiency and ion recoil velocity, simply by changing the wavelength of the ionization laser. While the 1 + 2′ REMPI scheme provides interesting prospects for high-resolution detection of NH scattering products, the ionization efficiency was found to be about a factor 20 lower than the 2 + 1 REMPI scheme results via the D state. Considering the low cross sections predicted for the NH-He system, the relatively low ionization efficiency was deemed insufficient to measure a large body of scattering data across a large collision energy window.

An alternative, remarkably efficient, ionization pathway was found in 3 + 1 REMPI detection using photons at a wavelength of around 336 nm. The resonant excited state could not be identified (and is marked with “?” in Figure 3) but lies just below the D state two-photon resonances in energy, and likely is of ^3^Σ character. The high ionization efficiency was attributed to the exceptionally strong A ^3^Π, *N*′ = 1, *j*′ = 2 ← X ^3^Σ^–^, *N*″ = 0, *j*″ = 1 transition, which is nearly resonant with a single 336 nm photon.^43^ To avoid confusion, we add an asterisk to the wavelength (336 *nm) to indicate that this 3 + 1 scheme involves a first color wavelength that differs slightly from the center frequency of the A ← X transition. For this scheme, a 7 mm diameter laser beam with a pulse energy of 10 mJ in a 5 ns pulse was produced using a frequency doubled dye laser (LiopTec) pumped by the second harmonic of an Nd:YAG laser (Innolas SL 1500). The laser beam was partially focused in the interaction region using a spherical lens with 470 mm focal length which was put 3 cm out of focus. The high power density used completely saturates the A ← X transition when on resonance and causes strong power broadening. The resulting spectrum is shown in Figure 4(b), in which the 2 + 1 REMPI spectrum from Figure 4(a) is shown again as an overlay. In order to facilitate the comparison, fundamental wavelengths for the involved lasers are used on the horizontal axis. The fundamental wavelength for the rR line of the one-photon A ← X resonance that addresses the population in the *N* = 0, *j* = 1 level is indicated by the vertical dashed line. Clearly, the main 3 + 1 REMPI line is slightly red-shifted with respect to the A ← X (rR) line. Moreover, the 3 + 1 REMPI scheme offers much higher signal levels than the 2 + 1 REMPI scheme with the available lasers. It is noted that, without identification of the excited electronic state, one cannot be sure about the state selectivity of this REMPI scheme at the full (*N*, *j*) level and its suitability for state-selective collision experiments. A proper spectroscopic investigation of this state is beyond the scope of our investigations.

To ensure state selectivity, a 1 + 2′ + 1′ REMPI scheme was developed as an extension to this new 3 + 1 scheme, where the first color laser (as before around 336 nm, 14.5 mJ) is now unfocused and tuned resonantly to the rR (*N*″ = 0, *j*″ = 1) line of the A ← X transition,^43^ and a second color (also around 336 nm, but slightly detuned from the first color wavelength) is used to further excite to the unidentified intermediate state and to ultimately ionize the molecule. For the second color excitation and ionization, a 7 mm diameter laser beam with a typical pulse energy of 15 mJ in a 5 ns pulse was employed, produced by the same laser system used in the (3 + 1) REMPI scheme described above. The second color laser was partially focused in the interaction region by a spherical lens with a 470 mm focal length that was put 3 cm out of focus. The arrival-time delay of the second laser was set to a few tens of nanoseconds with respect to the first laser to ensure proper overlap within the ∼450 ns lifetime of the A ^3^Π state.^42^ The resulting two-color spectrum was then used to obtain a difference spectrum by subtracting the spectrum observed when only the second color was used. This difference spectrum is shown in Figure 4(c), together with the one-color spectrum recorded with the second color laser only. With only the second color present, the NH radicals can only ionize through the 3 + 1 REMPI process mentioned before, and we retrieve a similar spectrum to the one presented in panel (b). By using two colors, and by fixing the first color to the A ^3^Π transition while scanning the second, only a single quantum state is probed. Note that, when the first color is present, the second color can still ionize NH radicals directly from the ground state via the 3 + 1 process. However, part of the NH radicals is already excited to the A-state by the first color, depleting the initial state, and thus resulting in a dip (negative signal) in the difference spectrum. All rotational transitions from the well-known A ← X electronic transition in NH can be used to probe individual (*N*, *j*) quantum states in the X ^3^Σ^–^ electronic ground state. This was verified by measuring individual spectra using the rR, rQ, rP, sR, and sQ branches as first color. As an example, the observed difference spectrum using the sR (*N*″ = 0, *j*″ = 1) line is shown in Figure 4(c) as well. Furthermore, He-seeded beams were used to increase the rotational temperature of the beam and to probe excited rotational levels relevant to inelastic scattering experiments, again investigating the use of various A ← X branches and corresponding second color resonances (data not shown).

The 1 + 2′ + 1′ scheme described above offers an estimated factor of 20 increase in ion yield with respect to 2 + 1 ionization via the D state. Unfortunately, the ion recoil of around 25 m/s using this scheme is not zero, but it is much smaller than the 38 m/s recoil imparted by the 2 + 1 REMPI scheme. Furthermore, the use of longer wavelengths, the enlarged ionization volumes, the possibility to cross the two laser beams in a well-defined volume, and the ability to tune the powers of both lasers independently together allow for an order of magnitude decrease in ionization of background gas, further improving the overall sensitivity. Efforts to alter this REMPI scheme to obtain nearly recoil-free detection using 1 + 3′ + 1′ or 1 + 2′ + 1″ variants remained unsuccessful.

### III.B Scattering Results

The 1 + 2′ + 1′ REMPI scheme was used to conduct state-to-state scattering experiments between NH (X ^3^Σ^–^, *N* = 0, *j* = 1) radicals and He atoms at various collision energies.

Detection of the NH (*N* = 2, *j* = 3) final state was chosen as the NH (*N* = 1, *j* = 1,2) channels suffered a significant contribution of elastic background signal, which could not be fully separated from the inelastic signal due to the significant ion recoil. The strong elastic contribution for these channels arises from a small amount of initial *N* = 1 molecules that is codecelerated with the *N* = 0 ground state, in combination with an unfavorable ratio of high elastic and low inelastic cross sections. The NH (*N* = 1, *j* = 0) exit channel should have a very low elastic background signal, as NH (*N* = 1, *j* = 0) molecules are defocused by the decelerator. However, the predicted inelastic cross section for the *N* = 0, *j* = 1 →*N* = 1, *j* = 0 channel is strongly reduced with respect to the other channels considered here,^18^ and very low scattering signals would be expected in our experiment. The NH (X ^3^Σ^–^, *N* = 2, *j* = 3) scattering products were probed using as the first color the A ^3^Π ← X ^3^Σ^–^ (sR) transition at 29991.34 cm^–1^ (∼14 mJ, unfocused), which ensures the selection of only the *j* = 3 spin-rotation level through the electric dipole transition selection rules.

Two types of collision experiments were conducted. In the first, we measured scattering images at selected collision energies ranging from ∼118 to ∼427 cm^–1^. For these measurements, the initial NH (X ^3^Σ^–^, *N* = 0, *j* = 1) velocity was kept constant (530 m/s guiding), while the mean He velocity was tuned by adjusting the ELV temperature between 50 and 300 K. In the second, we probed both the integral and differential cross sections in a relatively narrow range of collision energies around the opening of the *N* = 2, *j* = 3 channel at 97.7 cm^–1^. In these measurements, the temperature of the ELV (45 K) and resulting velocity of the He beam were kept constant. Instead, to obtain precise control over the collision energy, the pulsing sequence of the Zeeman decelerator was adjusted to vary the NH velocity from 300 to 650 m/s in steps of 25 m/s.

**Figure 5:**
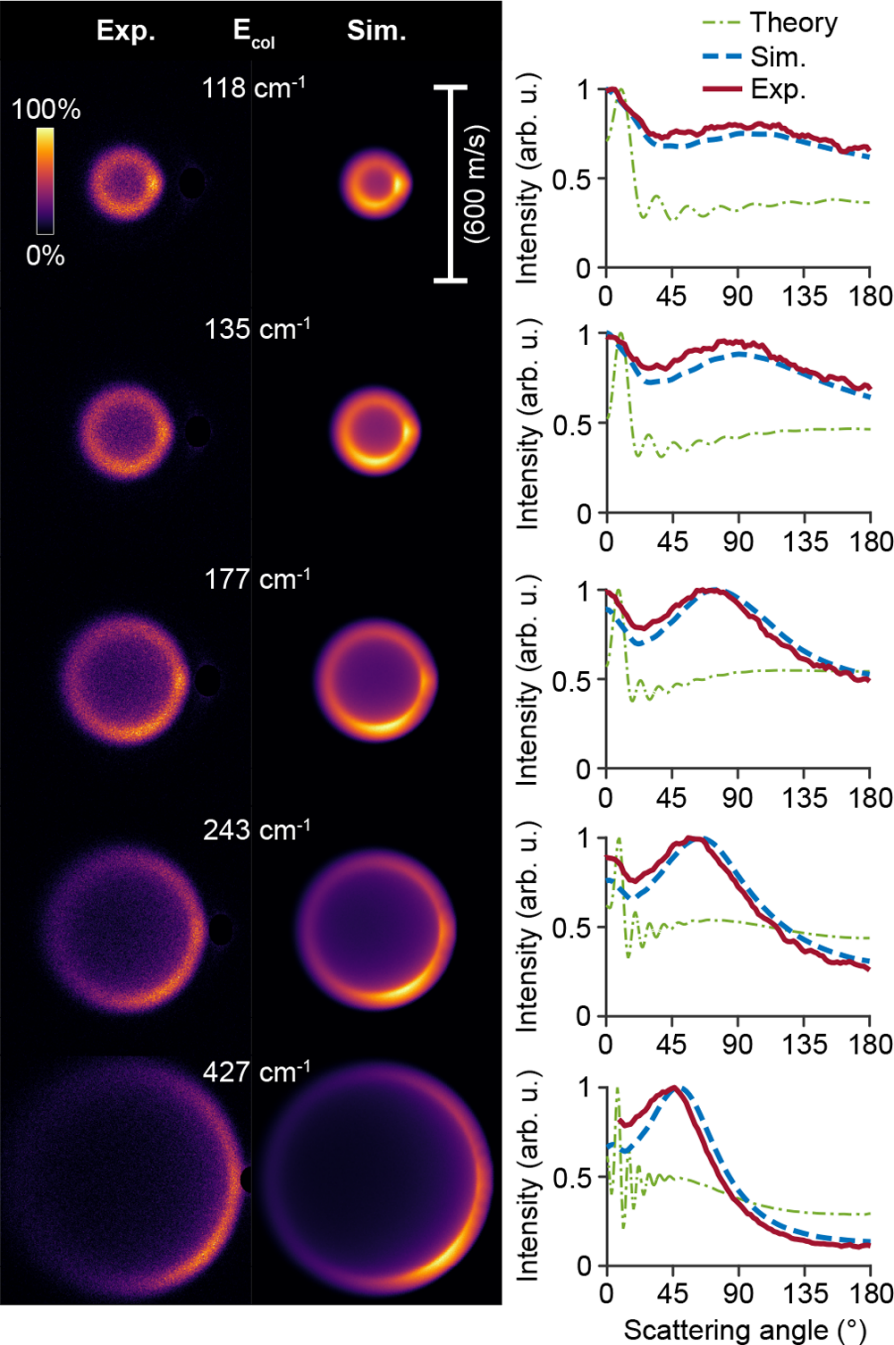
Experimental (Exp.) and simulated (Sim.) scattering images at selected collision energies (*E*_col_) for the NH (X ^3^Σ^–^, *N* = 0, *j* = 1) + He → NH (X ^3^Σ^–^, *N* = 2, *j* = 3) + He process, corresponding to an excitation energy of 97.7 cm^–1^. One image pixel corresponds to a velocity of 2.16 m/s. The angular scattering distributions extracted from the experimental (solid red lines) and simulated images (dashed blue lines) are shown in the right panels, together with DCSs from theory (dashed green lines). The different curves were normalized on the maximum intensity for clarity.

The images recorded for energies at and above ∼118 cm^–1^ are shown in Figure 5. The images are consistently presented such that the relative velocity vector is directed horizontally, with forward scattering angles positioned at the right side. The laboratory zero velocity point is located in the lower half of each image. Small segments of the images are masked where the initial beam gives a contribution to the signal. The collision energy was calibrated from the diameter of the recorded images. It is apparent that fine structures, like diffraction oscillations, cannot be discerned in these images due to the 25 m/s recoil velocity imparted to the ions during the ionization step. Yet, larger structures can be clearly identified, like the appearance of strong forward scattering at low collision energies and enhanced scattering intensity at side-scattered angles. The angular scattering distributions were extracted from the experimental image intensities within a narrow annulus around the observed rings. The experimentally obtained distributions can be directly compared to those obtained from simulated images. These image simulations are based on theoretical state-to-state differential cross sections acquired from *ab initio* quantum coupled-channels calculations that use state-of-the-art NH-He potential energy surfaces,^18^ in combination with the particle trajectory simulations on our Zeeman decelerator apparatus. In these simulations, we assume a constant DCS over the energy distribution sampled for a given experimental setting; this assumption is valid as the DCS is not expected to abruptly change within the collision energy distribution for a given setting of the experiment. Rapid changes in the DCS are only expected around pronounced scattering resonances and at lower collision energies. The simulated images take into account the spatial, temporal, and velocity spreads of the used molecular beams,^3^ as well as kinematic effects and the experimental ion recoil. The simulated images are analyzed analogously to their experimental counterparts to acquire predicted angular scattering distributions, of which the coarse shape may differ from the theoretical DCSs mainly due to more efficient detection of particles with low laboratory frame velocities after scattering.^3^ The measurements are found to be in good agreement with the simulated distributions.

**Figure 6:**
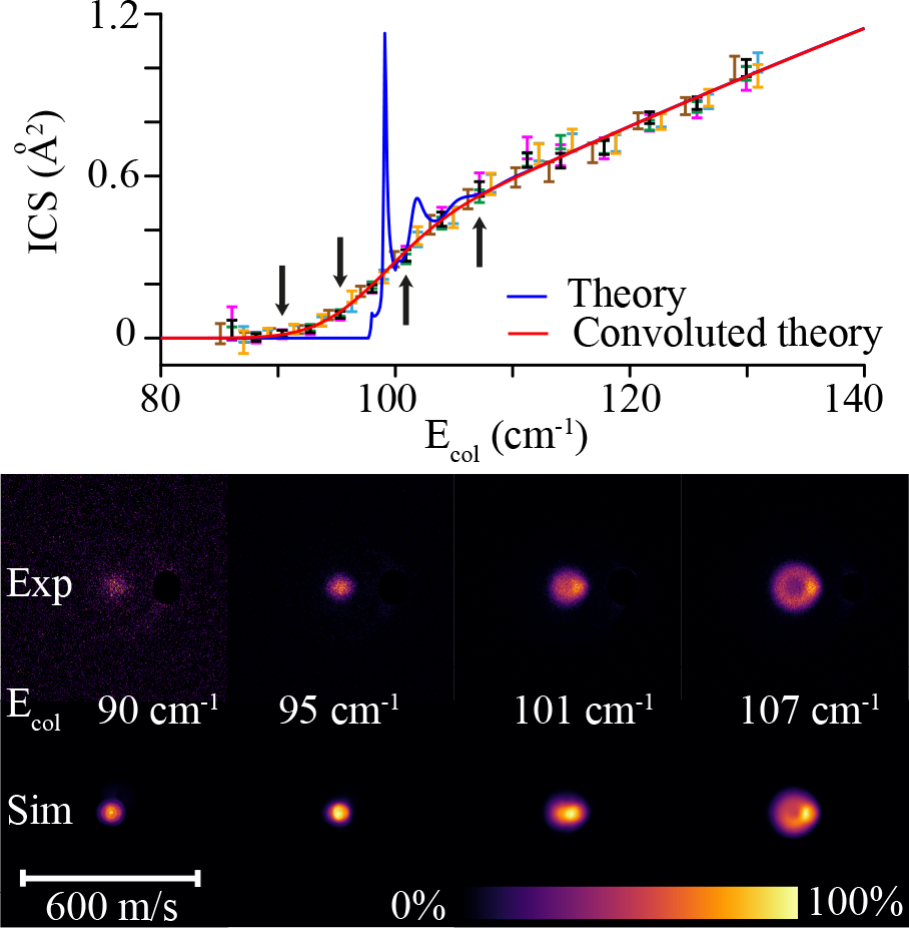
Experimental excitation function for the NH (X ^3^Σ^–^, *N* = 0, *j* = 1) + He → NH (X ^3^Σ^–^, *N* = 2, *j* = 3) + He process, corresponding to an excitation energy of 97.7 cm^–1^, together with the theoretical ICS (blue line) and the theoretical ICS convoluted with a collision energy spread of 10 cm^–1^ FWHM. The colored data points correspond to six different measurement sets that were each fitted to convoluted theory through the mean He velocity, collision energy spread, and a uniform intensity scaling parameter. The error bars represent the experimental uncertainty at 95% confidence interval. The vertical arrows indicate the collision energies at which scattering images were measured, which are shown together with simulated images in the lower panel. One image pixel corresponds to a velocity of 2.16 m/s.

The scattering images recorded at four energies around the channel opening are depicted in Figure 6. From these images, it is apparent that the ring size and signal intensity quickly drop with decreasing collision energy. The recorded images around the channel opening qualitatively agree with the simulated images based on theoretical DCS predictions. The behavior of the ICS around the channel opening was probed by rapidly cycling through the decelerator sequences corresponding to various NH velocities (300–650 m/s in steps of 25 m/s) while measuring the integral signal recorded in the relevant area of the camera. Figure 6 shows the relative integral cross section as a function of the collision energy, referred to as the excitation function, that is obtained after correction for background signals, variation of initial beam density, and a simplified density-to-flux transformation that is valid in the limit of small laser ionization volume in relation to beam overlap volume.^44^

For these near-threshold measurements, the images are too small to reliably calibrate the collision energies from the diameter of the images. The collision energy was more accurately calibrated by fitting the measured excitation function to the theoretical ICS prediction around the channel opening, using the He velocity (opening energy) and collision energy spread (opening shape) as fitting parameters. The measured ICS curves and corresponding fit parameters only slightly varied between measurement series, indicating a high stability of the experiment. A mean helium velocity of 750 ± 5 m/s and a collision energy spread of approximately 10 cm^–1^ (FWHM) were determined. The measured scattering probabilities are found to be in excellent agreement with the theoretical prediction after convolution with the deduced energy spread.

## IV Conclusion

We have presented the first state-to-state inelastic collision experiment using beams of Zeeman decelerated NH (X ^3^Σ^–^, *N* = 0, *j* = 1) radicals and tested the performance of various new REMPI schemes for NH in terms of sensitivity and ion recoil velocities compared to a conventional REMPI scheme’s performance. We found a highly efficient 1 + 2′ + 1′ REMPI scheme for the state-selective detection of NH and used it to perform the first crossed beam scattering experiment using Zeeman-decelerated NH, focusing on the NH-He system at relatively low collision energies. Although the 25 m/s ion recoil associated with this scheme does not allow for the observation of fine structures in the scattering images, it is ideally suited for ICS measurements and still allows for the observation of somewhat less delicate features in the scattering distributions. We probed differential cross sections for inelastic scattering in the 118–427 cm^–1^ energy range and probed both integral and differential cross sections at energies around the *N* = 0 → *N* = 2 channel opening at ∼98 cm^–1^. Excellent agreement was found with predicted cross sections from quantum scattering calculations using an accurate NH-He interaction potential.

Our search for new REMPI schemes was primarily aimed at the use of NH in controlled crossed-beam scattering experiments, but the efficient REMPI scheme presented here may, for example, find applications in experiments that aim for the trapping of NH after Zeeman deceleration as well. The scattering results on the NH-He system presented here demonstrate that our Zeeman decelerator can be applied in crossed-beam experiments to accurately probe both integral and differential cross sections, even for challenging scattering systems that involve low cross sections, inefficient discharge beam production, and intricate detection schemes.
